# Developing New Peptides and Peptide–Drug Conjugates for Targeting the FGFR2 Receptor-Expressing Tumor Cells and 3D Spheroids

**DOI:** 10.3390/biomimetics9090515

**Published:** 2024-08-27

**Authors:** Mary A. Biggs, Amrita Das, Beatriz G. Goncalves, Molly E. Murray, Sophia A. Frantzeskos, Hannah L. Hunt, Chau Ahn N. Phan, Ipsita A. Banerjee

**Affiliations:** Department of Chemistry and Biochemistry, Fordham University, 441 East Fordham Road, Bronx, NY 10458, USA; mbiggs2@fordham.edu (M.A.B.); adas21@fordham.edu (A.D.); bgoncalves1@fordham.edu (B.G.G.); mmurray76@fordham.edu (M.E.M.); sfrantzeskos1@fordham.edu (S.A.F.); hhunt7@fordham.edu (H.L.H.); cphan8@fordham.edu (C.A.N.P.)

**Keywords:** fibroblast growth factor receptor (FGFR2), tumor cell targeting, peptides, biomimetic, 3D spheroids, molecular dynamics, molecular docking

## Abstract

In this work, we utilized a biomimetic approach for targeting KATO (III) tumor cells and 3D tumoroids. Specifically, the binding interactions of the bioactive short peptide sequences ACSAG (A-pep) and LPHVLTPEAGAT (L-pep) with the fibroblast growth factor receptor (FGFR2) kinase domain was investigated for the first time. Both peptides have been shown to be derived from natural resources previously. We then created a new fusion trimer peptide ACSAG-LPHVLTPEAGAT-GASCA (Trimer-pep) and investigated its binding interactions with the FGFR2 kinase domain in order to target the fibroblast growth factor receptor 2 (FGFR2), which is many overexpressed in tumor cells. Molecular docking and molecular dynamics simulation studies revealed critical interactions with the activation loop, hinge and glycine-rich loop regions of the FGFR2 kinase domain. To develop these peptides for drug delivery, DOX (Doxorubicin) conjugates of the peptides were created. Furthermore, the binding of the peptides with the kinase domain was further confirmed through surface plasmon resonance studies. Cell studies with gastric cancer cells (KATO III) revealed that the conjugates and the peptides induced higher cytotoxicity in the tumor cells compared to normal cells. Following confirmation of cytotoxicity against tumor cells, the ability of the conjugates and the peptides to penetrate 3D spheroids was investigated by evaluating their permeation in co-cultured spheroids grown with KATO (III) and colon tumor-associated fibroblasts (CAFs). Results demonstrated that Trimer-pep conjugated with DOX showed the highest permeation, while the ACSAG conjugate also demonstrated reasonable permeation of the drug. These results indicate that these peptides may be further explored and potentially utilized to create drug conjugates for targeting tumor cells expressing FGFR2 for developing therapeutics.

## 1. Introduction

Current limitations associated with existing cancer treatment can span from side effects to lack of specificity and emerging drug resistance [1]. To address these inadequacies, several biomimetic approaches are being developed, including nanoscale theranostics [2], immunotherapy [3,4], aptamers [5] and dendrimers [6]. In particular, peptide-based tumor targeting has gained importance in recent years because of high biocompatibility and tumor targeting ability [7]. There have been several studies investigating a variety of short peptide sequences obtained from natural resources or from phage display libraries for targeting of overexpressed receptors in tumor cells [8,9,10]. For example, the sequence FIMGPY, derived from skate cartilage protein hydrolysate, was found to induce apoptosis in HeLa cells through caspase-3 activation [11]. In a separate study, the sequence YALPAH, isolated from the half-fin *Setipinna taty* anchovy, showed antiproliferative activity against human prostate cancer cells [12]. In addition, peptides such as CD44-binding peptide and fibronectin-derived heparin-binding peptide have been conjugated with polyethylene glycol diacrylate (PEGDA) hydrogel matrices and have provided insights about the mechanism of tumorigenesis and maintenance of cancer stem cells [13].

To gain further insight into designing peptide-based therapeutics and small molecule drugs, in silico methods such as molecular docking and molecular dynamics (MD) simulations are being utilized. For example, molecular docking studies of the cyclic octapeptide cyclosaplin have revealed high binding affinities toward tyrosine kinase receptors such as epidermal growth factor receptor (EGFR), vascular endothelial growth factor receptor (VEGFR2) and protein kinase B (PKB) [14]. Furthermore, high binding affinities were also observed toward apoptosis-related proteins, thereby implying that the peptides may play a role in apoptosis [15]. Studies have also shown that molecular dynamics simulation is a powerful tool utilized to predict specific receptor-binding ability and blood–brain barrier penetration capability of tumor homing peptides such as AngioPep2, tLyp1 (CGNKRTR) and CLT-1 [16,17]. In a separate study, in silico methods were also utilized to identify peptides derived from sea urchins to target receptors EGFR, BRAF V600E and PI3K [18]. Additionally, molecular modeling studies were utilized to demonstrate that the C-terminal portion of collagen XIX (NC1(XIX)), which is known to be an integral component of the basement membrane of vascular, mesenchymal, epithelial and neuronal cells, possesses anti-tumor activity upon release by plasmin, and that the activity was conformation-dependent. [19].

To further increase efficacy, targeted peptide sequences have also been conjugated to chemotherapeutic drugs. For instance, Wang and co-workers recently developed a paclitaxel (PTX) conjugated fused peptide TAR, comprised of a tumor-targeting peptide, A7R, and the cell penetrating peptide TAT to form self-assembled nanoparticles that were found to successfully endocytose and target the tumor site through the NRP-1 receptor [20]. In another study, the peptide–drug conjugate DOTATATE, composed of the cyclic peptide H-D-Phe-Cys-Tyr-D-Trp-Lys-Thr-Cys-Thr-OH covalently linked to the chelator Tyr3-octreotate, has been successfully utilized to target gastro-entero-pancreatic neuroendocrine tumors [21]. In a recent study, sequences containing the KFKT motif were attached to polyethylene glycol diacrylate (PEGDA) and self-assembled into nanoparticles to generate enzymatically cleavable nanoparticles for on-demand release of bone morphogenic growth factor and vascular endothelial growth factors [22].

**Figure 1:**
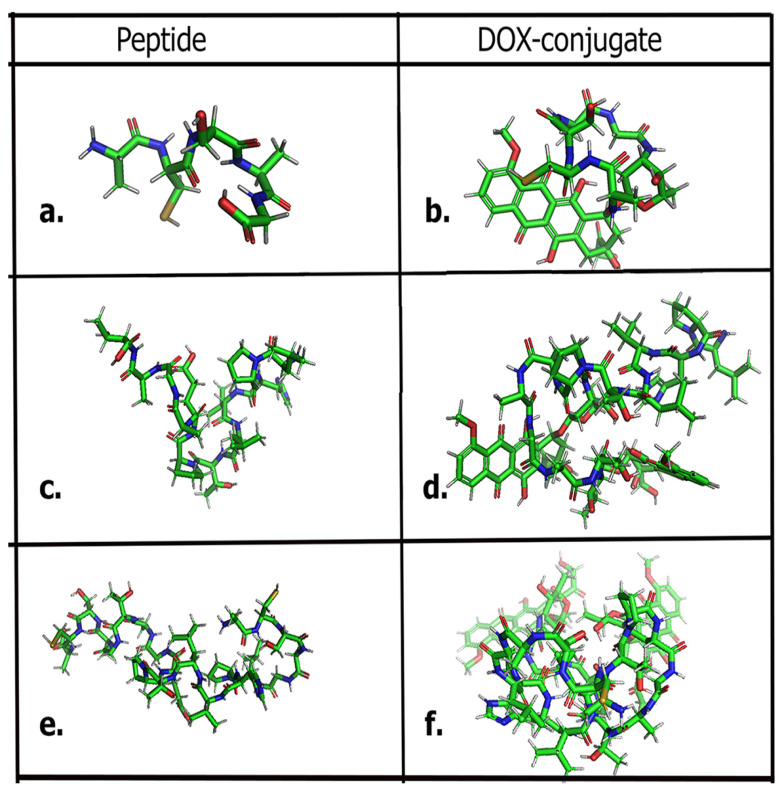
(**a**) ACSAG; (**b**) ACASA-DOX conjugate; (**c**) LPHVLTPEAGAT; (**d**) LPHVLTPEAGAT-(DOX)_2_ conjugate; (**e**) ACSAGLPHVLTPEAGATGASCA; (**f**) ACSAGLPHVLTPEAGATGASCA-(DOX)_2_ conjugate.

In this work, we utilized a biomimetic approach to investigate the FGFR2 kinase domain-binding activity of two short peptide sequences LPHVLTPEAGAT (L-pep) derived from dark muscle of tuna fish [23] and ACSAG (A-pep) [24], which was previously shown to be derived from earthworm species. We specifically examined FGFR2 because it is overexpressed in several types of cancer cells. In fact, FGFR2 gene amplification and gene alteration is one of the leading causes of gastric cancers, making FGFR2 a coveted target for the development of therapeutics [25]. In addition to the above two peptide sequences, we created a third amphiphilic peptide sequence by integrating both of these peptides into a single fused peptide sequence to explore if the targeting ability of the peptides may be enhanced. Thus, the peptide ACSAGLPHVLTPEAGATGASCA (Trimer-pep) was designed. In particular, FGFR2 overexpression is highly prevalent in gastric, renal, colorectal and breast tumor cells [26,27,28]. Previous studies have shown that LPHVLTPEAGAT can target MCF-7 breast cancer cells and reduce proliferation; however, to our knowledge, the mechanism has yet to be explored. The ACSAG peptide, on the other hand, has been found to exert antimicrobial effects; however, the ability of this peptide to target tumor cells has never been studied. In a recent study, it was demonstrated that several bioactive antimicrobial earthworm-derived peptides inhibited the proliferation of tumor cells [29]. We hypothesized that given the structural motifs of the peptides, they may potentially bind to the kinase domain of the FGFR2. Specifically, the binding site of FGFR2 includes a highly conserved charged residue Asp644, which is part of the DFG motif and has been shown to form hydrogen bonds with existing FGFR inhibitors such as the pyrazole derivative AZD4547 [30]. Another highly conserved residue, Cys491, from the glycine-rich loop region, has been shown to interact with FGFR2 inhibitors such as 3,5-disubstituted indolin-2-one derivatives [31]. Given that the peptides ACSAGLPHVLTPEAGATGASCA, ACSAG and LPHVLTPEAGAT are amphipathic and contain residues capable of forming hydrogen bonds as well as hydrophobic interactions, we examined the binding interactions of these peptides with the FGFR2 kinase domain (residues 461–763) through molecular docking studies and through MD simulations. Furthermore, the presence of cysteine in the peptides may enhance interactions. To create drug delivery agents, each of these peptides were conjugated to the chemotherapeutic drug doxorubicin (DOX) to increase efficacy. Overall, in silico results indicated that the peptides, particularly ACSAGLPHVLTPEAGATGASCA and ACSAG, showed stable binding toward FGFR2, and conjugating with DOX further increased their binding affinities. The chemical structures of the peptides and the DOX conjugates are shown in Figure 1.

As proof of concept, following in silico *studies*, we examined the tumor-targeting ability of the peptides and peptide–drug conjugates with gastric tumor cells (KATO III), which overexpress the FGFR2 receptor. In addition, SPR analysis was carried out to validate the binding interactions of the peptides with the FGFR2 protein kinase domain. Furthermore, to mimic the 3D tumor microenvironment, 3D spheroids comprised of co-cultured colon tumor-associated fibroblasts (CAFs) and gastric cancer cells were grown in order to examine if the peptide–drug conjugates could penetrate the 3D spheroids. CAFs are known to particularly accelerate tumor progression and metastasis by modifying the extracellular matrix of cells, and are therefore considered to be viable targets [32]. Results indicated that higher cytotoxicity was observed for the tumor cells compared to normal fibroblasts. Furthermore, the peptides ACSAG and ACSAGLPHVLTPEAGATGASCA and their drug conjugates were found to permeate deeper into the spheroids compared to the neat drug. Thus, these sequences may be further potentially developed for developing tumor-targeted therapeutics for tumor cells overexpressing the FGFR2 receptor.

## 2. Materials and Methods

### 2.1. Materials

The peptide sequences A-C-S-A-G, L-P-H-V-L-T-P-E-A-G-A-T, and A-C-S-A-G-L-P-H-V-L-T-P-E-A-G-A-T-G-A-S-C-A were custom-ordered from GenScript. Doxorubicin was purchased from Cayman Chemical Company (Ann Arbor, MI, USA). N-hydroxysuccinimide (NHS), 1-ethyl-3-(3-(dimethylamino)propyl) carbodiimide (EDAC), and dimethylformamide (DMF) were purchased from Sigma-Aldrich (St. Louis, MO, USA). Fetal bovine serum (FBS), phosphate-buffered saline (PBS), antibiotic–antimycotic mixture, Iscove’s Modified Medium (IMDM) with L-glutamine, human lung fibroblast cells and KATO III human gastric carcinoma cells (HTP-103), were purchased from ATCC (Manassas, VA, USA). Human colon tumor-associated fibroblasts (HC-6231) were purchased from Cell Biologics (Chicago, IL, USA). Trypsin-EDTA 1× and Nunclon Sphera 12-well and 96-well plates were purchased from ThermoFisher Scientific. The human FGFR2 protein (His-tag) residue 403–822 was purchased from Thermo Fisher Scientific. The FGFR2 ELISA kit was purchased from Sino Biological. WST-8 assay reagent was bought from Cayman Chemicals. Alexa Flour 647 FGFR2 antibody was purchased from Santacruz Biotechnology, Dallas, TX, USA. Gold biosensor chips (SF-10 glass, index = 1.72) were ordered from Platypus Technologies, and index fluid was purchased from Cargille Labs, Cedar Grove, NJ, USA.

### 2.2. Methods

#### 2.2.1. Computational Methods

##### Peptide Design

The peptides A-C-S-A-G, L-P-H-V-L-T-P-E-A-G-A-T, and A-C-S-A-G-L-P-H-V-L-T-P-E-A-G-A-T-G-A-S-C-A and their DOX conjugates were drawn using ChemDraw 22.2, followed by MM2 energy minimization and conversion to .pdb files using Chem3D. Each of the .pdb files was visualized in PyMOL v. 2.4.0 [33]. To create conjugates, the free carboxyl groups of the peptides were conjugated with the free amine group of doxorubicin. In the case of L-P-H-V-L-T-P-E-A-G-A-T and A-C-S-A-G-L-P-H-V-L-T-P-E-A-G-A-T-G-A-S-C-A, di-conjugates were created due to the presence of glutamic acid residue in addition to the C-terminal carboxyl group, while a monoconjugate of DOX was created for ACSAG.

##### Anti-CP Studies

To predict the anticancer potential of the peptides, the webserver AntiCP was used [34]. This software utilizes the amino acid composition of the peptides, and binary profile features such as k-space and the position-specific scoring matrix, and evaluates them by machine learning methods, such as the support vector machine (SVM) and sequential minimal optimization (SMO), to build models to differentiate ACPs from whole peptides. A second model that distinguishes anticancer peptides from antimicrobial peptides based on datasets was also utilized. Furthermore, it also provides information about physicochemical properties such as hydropathicity, pI, amphipathicity, hydrophobicity and hydrophilicity.

##### Binding Pocket Analysis

Binding pocket analysis was performed on the receptor with the Pocket-Cavity Search Application (POCASA) web server [35]. The X-ray crystal structure .pdb file of the kinase domain dimer of FGFR2 was downloaded from the RCSB Protein Data Bank, and water molecules and any attached ligands were removed using PyMOL. The PDB ID: 7OZY [36] was used. This webserver examines protein structures by creating a 3D grid system containing the atoms of the receptor. The program utilizes the “Roll” algorithm to identify potential ligand-binding pockets and cavities from the 3D structure that was created.

##### Molecular Docking Studies

Molecular docking studies were conducted using Autodock Vina v. 1.5.7 [37,38]. Receptor and ligand files were prepared and saved in .pdbqt format using Autodock Tools. System-determined docking grids (dimensions 40 Å × 40 Å × 40 Å) were used for the receptors. The coordinates of the grid box were (x = 24.685, y = −10.098, z = 115.331). Exhaustiveness and energy range were kept at default values of 8 and 4, respectively. Docking studies were then carried out for each of the designed peptide sequences and drug conjugates with the receptor using Autodock Vina to determine binding affinities and optimal docking configurations. The output .pdbqt files obtained for the highest-ranked models were downloaded and visualized on PyMOL, and the corresponding binding affinities were recorded.

##### Protein–Ligand Interaction Profiler (PLIP) Analysis

To characterize the binding interactions, optimal binding poses of the receptor–ligand configurations produced using Autodock Vina were uploaded to the PLIP online interface [39]. The output .pdbqt files generated from Autodock Vina and the receptor .pdbqt file were opened in PyMOL (2.4.0) and exported as a single .pdb file. This .pdb file was then uploaded onto the PLIP web server. PLIP generated results in .pse and .txt formats. Both formats included the receptor residues involved in interactions and the distance of the bonds. The results were tabulated, visualized and analyzed.

##### Molecular Dynamics

Receptor–ligand molecular dynamics simulations were conducted using GPU-accelerated Desmond software through Maestro version 2023-2 [40,41]. For each protein–ligand complex, the output .pdbqt binding pose and the receptor .pdb files were opened in PyMOL and exported as a single .mae file. This .mae file was then opened in Maestro for preparation for molecular dynamics simulation studies. Using the Protein Preparation Wizard application in Maestro, hydrogens and missing side chains were added to the receptor. Hydrogen bonds were optimized at a pH of 7.4, and restrained minimization was carried out. Heavy atoms were converged to 0.30 Å RMSD. Using the System Builder application in Maestro, a 10 Å × 10 Å × 10 Å grid box was formed around the protein–ligand complex with the SPC solvent model and OPLS-2005 force field. This force field builds on prior versions to add additional modeling parameters, thereby improving the accuracy of complex simulations [42]. To mimic physiological conditions, the complex was surrounded by water, and the aqueous system was neutralized with sodium and chloride ions. In the Molecular Dynamics application, the run time was set to 100 ns with 1000 frames. An NPT ensemble class was selected to equilibrate the system at 300 K and 1 atm, followed by relaxation of the system through minimization prior to the start of the run. The prepared receptor–ligand complex was then loaded on DESMOND. Following completion of each run, the –out.cms file was loaded into Maestro. Protein–ligand RMSD and C-Alpha RMSD data as well as protein and ligand RMSF data were generated using the Simulation Interaction application for all complexes. Trajectory images at different time points in molecular dynamics simulations were analyzed to visualize binding interactions over time.

##### MM-GBSA Analysis

We utilized the Molecular Mechanics Generalized Born Surface Area (MM-GBSA) method to generate the relative binding free energies and evaluate the theoretical free energies of binding of the peptides and drug conjugates with the FGFR2 kinase domain. Output trajectory files from each MD simulation were visualized and analyzed using the script thermal_mmgbsa.py. The average free energy over the 100-nanosecond simulation was determined as representative of binding strength for each receptor–ligand complex. The Prime module of the Schrodinger Suite 2023-2 was used to calculate the free energies [43,44]. The complex free energy was calculated as ∆G bind = ∆G solv + ∆E MM + ∆G SA, where ∆G solv indicates the change in solvation energy of the protein–ligand complex from the summed individual solvation energies of the ligand and protein. ∆G SA is the difference in the energy of the surface area of the complex and the summed protein and ligand individually. ∆E MM is the difference in minimized energy between the complex and the summed individual protein and ligand [45]. The components contributing to free energy were averaged, tabulated and reported.

#### 2.2.2. Laboratory Analysis

##### Surface Plasmon Resonance

In order to validate computational results, surface plasmon resonance (SPR) studies were conducted. SPR analysis allows for the examination of live binding between antibodies or ligands with specific proteins [46]. In our studies, SPR was used to investigate the binding interactions of the three peptides as well as that of the known FGF Receptor Tyrosine Kinase Inhibitor—CAS 192705-79-6 (Santacruz Biotechnologies, Dallas, TX, USA) with the kinase domain of FGFR2. Analysis was performed using a GW- Imager, Surface Plasmon Resonance instrument. Gold chips (Platypus technologies, Madison, WI, USA) were first functionalized with 11-mercaptoundecanoic acid (1 M) solution prepared in ethanol, and were allowed to incubate for one hour. The chips were then coated with NHS (0.01 M) followed by EDAC (0.01 M) and then incubated for two hours at 4 °C in a petri dish. The chips were then coated with FGFR2 solution (10 µg/mL). These chips were allowed to incubate for 8 h at 4 °C, after which they were placed on the flow sensor, gold-face down, and a drop of 7.21 Cargille’s index fluid was added to the glass face before the prism was loaded. The instrument was allowed to calibrate with 1X PBS for 1 h. At the beginning of each run, the instrument was allowed to run for 100–200 s with 1X PBS before the analyte (specific peptide or known inhibitor) was injected. The peptides and the known inhibitor were then allowed be run through the receptor-bound chip at five different concentrations (50 nM, 100 nM, 1 µM, 50 µM and 100 µM). The analyte was allowed to run for 2000 s before switching to 1X PBS. The flow rate was kept constant at 30 µL/min. Following completion of each experiment, chips were washed by first soaking in 3:1 sulfuric acid and hydrogen peroxide solution (Pirahna solution) for 10 min, followed by rinsing with distilled water and washing with a 70% ethanol solution, and finally irradiating with UV light for 10 min. The data from the SPR studies were then input into GraphPad Prism 9.5 (Graphpad Software Inc., San Diego, CA, USA) to perform non-linear regression analyses in order to determine the K_D_ values for each sample. The values obtained for three separate runs for each sample were averaged and reported. Statistical analysis was carried out using Student’s *t* tests.

##### Peptide Conjugation with Doxorubicin (DOX)

The peptide–DOX drug conjugates were prepared using previously established peptide coupling methods [47]. Each peptide was first dissolved in DMF to 25 mg/mL concentration. In order to activate the carboxylic acid group on the peptide, NHS (10 mg/mL) and EDAC (10 mg/mL) were added. The solution was mixed for 1 h at 4 °C. Then DOX was added (25 mg/mL) and allowed to shake at 400 rpm for 48 h at 4 °C. In the case of the L-pep and Trimer-pep, di-conjugates were prepared, due to the presence of an additional free carboxyl group of those peptides. Thus, the DOX-to-peptide ratio was kept at 2:1 for those two conjugates. The solvent was rotary evaporated. The resulting product was recrystallized from (3:1) acetone-water and dried using a speedvac concentrator. Formation of the drug conjugates was confirmed using ^1^H NMR spectroscopy and Fourier Transform Infrared Spectroscopy (FTIR). For ^1^H NMR, samples were prepared using DMSO-d6 solvent that contained 0.3% TMS and analyzed using a Bruker 400 MHz NMR spectrophotometer.

For the ACSAG-DOX (A-pep-DOX) conjugate, peaks were seen at δ 0.98 (3H, d); δ 1.21 (3H, d); δ 1.32 (1H, s); δ 1.56 (3H, d); δ 1.98 (2H, d); δ 2.23 (2H, d); δ 2.92 (2H, d); δ 3.13 (2H, s); δ 3.53 (2H, m); δ 3.69 (1H, m); δ 3.82 (3H, s); δ 4.11 (2H, s); δ 4.20 (2H, d); δ 4.29 (1H, m); δ 4.46 (1H, t); δ 4.55 (1H, t): δ 4.61 (2H, m); δ 4.70 (2H, s); δ 4.82 (1H, t); δ 4.92 (1H, s); δ 5.10 (1H, s); δ 5.41 (1H, s); δ 7.21 (1H, d); δ 7.64 (1H, d); δ 7.69 (1H, d); δ 8.12 (1H, s); δ 8.31 (4H, s); δ 8.89 (2H, s); δ 11.92 (2H, s).

For the LPHVLTPEAGA-(DOX)_2_ (L-pep-(DOX)_2_) conjugate, peaks were seen at δ 0.92 (12H, d); δ 0.98 (6H, d); δ 1.13 (3H, d); 1.22 (3H, d); δ 1.35 (2H, d); δ 1.44 (6H, d); δ 1.52 (1H, m); δ 1.82 (3H, m); δ 1.96 (4H, m); δ 2.04 (2H, t); δ 2.12 (6H, m); δ 2.20 (4H, d); δ 2.35 (4H, m); δ 2.64 (1H, m); δ 2.96 (2H, d); δ 3.15 (1H, t); δ 3.40 (4H, d); δ 3.55 (2H, m); δ 3.62 (2H, m); δ 3.92 (6H, s); δ 4.10 (2H, s); δ 4.20 (2H, d); δ 4.31 (1H, d); δ 4.42 (2H, t); δ 4.49 (3H, m); δ 4.61 (2H, t); δ 4.68 (4H, m); δ 4.75 (4H, s); δ 4.83 (2H, s); δ 4.95 (3H, m); δ 5.52 (3H, s); δ 7.41 (2H, d); δ 7.65 (1H, s); δ 7.81 (2H, d); δ 7.88 (2H, d); δ 8.12 (2H, s); δ 8.33 (7H, s); δ 8.50 (1H, s); δ 8.82 (1H, s); δ 9.2 (1H, s); δ 11.8 (4H, s); δ 12.5 (1H, s).

For the ACSAG-LPHVLTPEAGA-GASCA-(DOX)_2_ (Trimer-pep-(DOX)_2_) conjugate, peaks were seen at δ 0.88 (12H, d); δ 1.01 (6H, d); δ 1.12 (3H, d); δ 1.25 (3H, d); δ 1.37 (2H, s); δ 1.46 (12H, d); δ 1.55 (2H, m); δ 1.81 (4H, d); δ 1.89 (4H, d); δ 1.96 (4H, m); δ 2.10 (4H, m); δ 2.34 (4H, d); δ 2.42 (4H, m); δ 2.62 (1H, q); δ 3.15 (4H, d); δ 3.33 (4H, d); δ 3.46 (2H, t); δ 3.55 (2H, t); δ 3.64 (1H, q); δ 3.80 (4H, q); δ 3.95 (6H, s); δ 4.11 (6H, s); δ 4.20 (4H, d); δ 4.28 (2H, d); δ 4.39 (1H, t); δ 4.45 (6H, t); δ 4.50 (2H, t); δ 4.57 (2H, t); δ 4.65 (8H, m); δ 4.72 (4H, s); δ 4.88 (4H, m); δ 5.1 (6H, s); δ 5.5 (3H, s); δ 6.1 (1H, s); δ 7.2 (2H, d); δ 7.5 (1H, s); δ 7.92 (4H, m); δ 8.15 (2H, s); δ 8.52 (14H, s); δ 8.72 (1H, s); δ 8.83 (2H, s); δ 9.2 (3H, s); δ 11.92 (2H, s); δ 12.6 (1H, s).

##### 2D Cell Cultures

Three cell lines were cultured in 50 mL sterile falcon tissue culture flasks. These included the FGFR2-expressing KATO III gastric cancer cell line (ATCC HTB-103), human colon cancer-associated tumor fibroblasts (HC-6231, Cell Biologics), and primary lung fibroblasts (normal) as control (ATCC-PCS-201-013). Both lines of fibroblasts were grown in Fibroblast Growth Media, and KATO III cells were cultured in Iscove’s Modified Dulbecco’s Medium (IMDM). All growth media were supplement with 10% fetal bovine serum (ATCC), 3 mL of 1X antibiotic-antimycotic mixture per 500 mL medium (GIBCO), 10 units/mL penicillin and 10 μg/mL streptomycin (GIBCO). Each cell line was incubated at 37 °C in a 5% CO_2_ incubator. Cells were grown to confluence, and the medium was changed every 2 to 3 days. Confluent cells were split twice a week.

##### Cytotoxicity Studies

Cells were plated in 96-well plates at a density of 1 × 10^4^ cells/well. WST-8 assays (Cayman Chemicals, Ann Arbor, MI, USA) were conducted in triplicate for non-cancer primary fibroblasts, as well as for KATO III cells, following manufacturer instructions from the assay kit. In general, varying concentrations of the peptides and the DOX conjugates were utilized. The concentrations used were 0.1 µM, 0.5 µM, 1 µM, 2 µM and 5 µM. Neat DOX (1 µM, 2 µM) was also compared. The peptides or drug conjugates or DOX were allowed to incubate with cells for 24 h, and imaged using a phase contrast optical microscope before analysis. Following incubation with WST-8 reagent according to manufacturer instructions, absorbance at 450 nm was measured using a Biotek microplate reader. The absorbance of DOX in media was subtracted.

##### ELISA

Because KATO III gastric cancer cells overexpress the FGFR2 receptor [48], we conducted ELISA assays to measure the FGFR2 levels for untreated KATO III gastric cancer cells according to the manufacturer’s instructions. Cells were plated at a density of 1 × 10^5^ cells per well and allowed to spread in 96-well plates for 48 h. Prior to the assay, solutions were prepared as per manufacturer instructions. Briefly, the wash buffer solution was diluted with deionized water to prepare 1X wash buffer. To prepare 1X dilution buffer, the 20X concentrate was diluted 20-fold with deionized water as required. To prepare the detection antibody, the stock provided was diluted at 0.25 µg/mL in 1X dilution buffer. To prepare the standard stock solution, 1 mL of 1X dilution buffer was added to the provided bottle of standard solution. To prepare the substrate solution, color reagents A (hydrogen peroxide solution) and B (tetramethylbenzidine, TMB) were combined in equal volumes 10 min before use. Then, 100 µL of cell culture supernatant was collected from the cultures. Following an initial wash of the sample wells with wash buffer, the supernatant was added to respective FGFR2 antibody-coated wells provided by the manufacturer. A standard curve was generated using the results obtained for the standard FGFR2 solution provided, with eight serial dilutions in the range of 0 pg/mL to 700 pg/mL. Following the addition of standards and experimental samples, the assay wells were incubated for 2 h at room temperature with gentle shaking. Then, 300 µL of wash buffer was added to each well and decanted after two minutes. This was repeated thrice. Next, the wells were incubated with 100 µL of the prepared detection antibody and incubated at room temperature for one hour with shaking. The solution was decanted and again washed four times. Then, 100 µL of the prepared substrate solution was added to each well. The wells were incubated for twenty minutes at room temperature in the dark and shaken gently. Finally, 100 µL of stop solution was added to each well, leading to the color change. The plate was mixed by gentle tapping. To read the results, the absorbance of the plate was read at 450 nm using a BioTek microplate reader.

##### Immunofluorescence Studies

KATO (III) cells were grown to confluence as described earlier. The confluent cells were plated at a cell density of 1 × 10^6^ cells/well in 24-well plates and allowed to spread for 72 h. Then, each of the peptides, at a concentration of 1 μM, was added to selected wells. An equivalent amount of deionized water was added to the cell controls and incubated for 24 h. Then the media was removed, and replaced with PBS. Cells were allowed to incubate for 4 h with the Alexa Flour 647-tagged FGFR2 antibody at a concentration of 5.0 ug/mL. After 4 h, the cells were imaged using a BioTek Cytation C10 confocal microscope (Agilent Technologies, Santa Clara, CA, USA). Both phase contrast and fluorescence images were taken and superimposed to obtain images showing cells that were stained with the antibody. Each study was done in triplicate.

##### Flow Cytometry

KATO (III) cells were plated at a density of 1 × 10^6^ cells/well in 24 well plates. After twenty-four hours, each of the peptides, at a concentration of 1 μM, was added to selected wells. An equivalent amount of deionized water was added to the cell controls and incubated for 24 h. Then the cells were allowed to incubate for 4 h at 37 °C in a CO_2_ incubator with the Alexa Flour 647-tagged FGFR2 antibody at a concentration of 5.0 ug/mL. The media was then removed, and wells were washed with PBS and then trypsinized. Following trypsinization for three minutes, IMIM media was added, and the cells were centrifuged to remove excess media. Cells were then washed and centrifuged twice with ice-cold FACS buffer (BD Biosciences). Samples were filtered into cell culture tubes and analyzed using a FACS melody instrument (BD Biosciences, Franklin Lakes, NJ, USA). Samples were excited at Cy5 wavelength (Emission max 676 nm). In general, the number of events was kept constant at 10,000 for all samples. Gating and further analysis was conducted using FlowJo v10.9.0 software.

##### Spheroid Growth

To mimic the tumor microenvironment, 3D cultures of tumor cells (spheroids) were grown. For growing spheroids, co-cultures of human colon-associated tumor fibroblasts and KATO III gastric cancer cells were grown in a 1:3 ratio in a growth media containing IMDM and fibroblast growth medium. Spheroids grown at 2000 cells/well and 4000 cells/well were co-cultured in Nunclon Sphera nonadherent 12-well plates and allowed to grow over a period of two weeks. The growth of the spheroids was monitored using optical microscopy. Media was cautiously removed every two days, and replaced with new medium.

##### PicoGreen Double Stranded (ds) DNA Assay

To further confirm the growth of the spheroids over time, we conducted PicoGreen DNA Assays to determine the DNA content of the spheroids over time [49]. Co-cultures of spheroids were grown in Nunclon Sphera nonadherent 12-well plates, and the amount of ds DNA was quantified at different time points for spheroids grown at 2000 cells/well and 4000 cells/well. To obtain DNA content, the spheroids were first lysed. To accomplish this, growth media was removed from the samples after 3 days of growth and after 10 days of growth. Samples were then washed with PBS to remove any residual growth medium to avoid any signal interference. This wash was then aspirated from the wells. Next, 350–400 µL of mammalian cell lysis buffer was added to each sample, and the plate was shaken gently at room temperature for 10 min. Following this incubation, samples were sonicated using a Fisher brand Model 505 sonic dismembrator at 55 Joules for five minutes. Based on the absorbance observed at 260 nm, the cell lysate samples were diluted using 50 µL of sample lysate solution with 2000 µL of TE buffer (10 mM Tris-HCl, 1 mM EDTA, pH 7.5). The stock was diluted to 0.04 absorbance at 260 nm using TE buffer, corresponding to a 2 µg/mL dsDNA stock solution, followed by serial dilutions for the preparation of the standard curve. Having prepared the samples and stock solutions, 100 µL of each was added to respective wells in the 96-well plate. Next, 100 µL of aqueous Quant-iT PicoGreen dsDNA reagent was added to each sample and standard solutions. Wells were incubated for 5 min at room temperature in the dark. Finally, fluorescence was measured by exciting at 480 nm and at an emission wavelength of 520 nm using a Biotek Synergy HT microplate reader. The DNA content of the samples was determined based on the standard curve plotted using serial dilutions.

##### Impact of Peptides and Peptide–Drug Conjugates on the Spheroids

The co-cultured spheroids were grown at a density of 2000 cells/well for six days prior to treatment with the peptides or the peptide–drug conjugates. To visualize the internalization of the peptides, those were first tagged with fluorescein to allow for visualization through fluorescence microscopy. For tagging, each peptide was tagged with NHS-Fluorescein (5/6-carboxyfluorescein succinimidyl ester to allow for binding with the amine group of the peptides). To each peptide solution (10 µM), 1 µM of the dye solution was added and allowed to shake in the dark for 24 h. The solutions were then centrifuged and washed with deionized water twice to remove any unbound dye. Then, peptide solutions were vortexed and allowed to incubate with the spheroids. For the drug conjugates, no tagging was done, due to inherent fluorescence of DOX. In general, 1 µM solutions of the peptides or drug conjugates were used. The spheroids were then visualized using fluorescent microscopy using an inverted Amscope IN480TC-20MB13 microscope at various magnifications.

##### SEM Imaging of Spheroids

The co-cultured spheroids were allowed to grow for ten days as described above. To prepare samples for SEM imaging, media was removed from each well, followed by washing with phosphate-buffered saline (PBS). Each spheroid was then carefully transferred to a silicon chip precoated with poly-L-lysine in a 6-well plate. Following placement, the spheroid samples were fixed with 2.5% gluteraldehyde in pH 7.4 sodium cacodylate (0.1 M). This solution was allowed to incubate at room temperature for 1 h. The samples were then rinsed three times in 0.1 M sodium cacodylate buffer and allowed to dry for 30 min at room temperature. The samples were then incubated in 1% osmium tetroxide for 2 h. The samples were finally dried with a sequence of ethanol solutions of increasing concentrations: 10%, 25%, 50%, 70% and 95% *w*/*w*.

### 2.3. Characterization

#### 2.3.1. Fluorescence Imaging

To visualize the internalization of the peptides and the peptide–drug conjugates in the spheroids, we conducted fluorescence microscopy using an Amscope Inverted Phase-Contrast Fluorescence Microscope. Samples were imaged at various magnifications.

#### 2.3.2. SEM Imaging

Spheroids were imaged using a Zeiss EVO MA10 model scanning electron microscope. Samples were examined at a range of 5 to 10 kV at varying magnifications. In general, the instrument was operated in EP mode.

#### 2.3.3. Fourier Transform Infrared (FTIR) Spectroscopy

Fourier transform infrared spectroscopy was conducted to confirm the conjugation of DOX with the peptides using a Thermo Scientific Nicolet IS50 FTIR (Thermo Scientific, Waltham, MA, USA) with OMNIC Software (Thermo Scientific, Waltham, MA, USA). KBr pellets of samples were prepared, and all spectra were taken at 4 cm^−1^ resolution with 100 scans for averaging. The sample measurements were taken between 400 and 4000 cm^−1^.

#### 2.3.4. Differential Scanning Calorimetry (DSC)

To examine thermal phase changes of the peptides and the drug conjugates, DSC analyses were carried out using TA Instruments Q200 DSC (TA Instruments, New Castle, DE, USA). Studies were carried out in the range of 0 to 250 °C under nitrogen flow at the rate of 10 °C per minute. Samples were dried under vacuum prior to analysis. In general, samples in the range of 1.5 to 2 mg were used. Each study was carried out in triplicate.

## 3. Results and Discussion

### 3.1. Anti-CP Studies

In order to predict the anticancer potential of the peptides, each of the sequences were uploaded onto the Anti-CP webserver. The SVM scores and the properties of each of the peptides are shown in Table 1. Interestingly, the anti-CP score given by the machine learning algorithm according to the prediction model was found to be the highest for the ACSAG (A-pep) while those obtained for LPHVLTPEAGAT (L-pep) and ACSAGLPHVLTPEGATGASCA (Trimer-pep) were comparable. Furthermore, all three peptides were predicted to be anti-CP. In previous studies, it has been shown that peptides with SVM scores greater than 0.5 are considered to have active anticancer properties [50]. Thus, it is anticipated that the three peptides utilized may have potential in targeting tumor cells. In addition, the pI values of the peptides were found to be in mildly acidic range, while the hydrophobicity was found to be higher for the ACSAG peptide.

### 3.2. Binding Pocket Analysis

**Figure 2:**
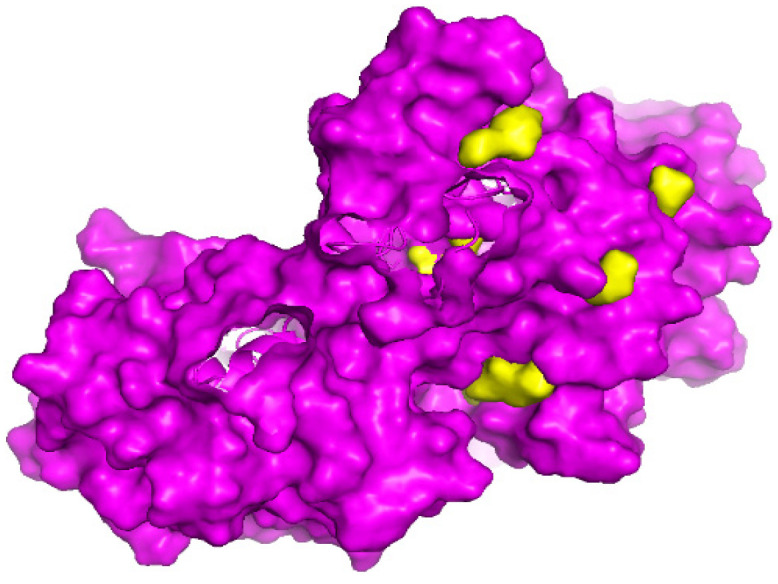
Binding pockets within the FGFR2 kinase domain dimer. Binding pockets are shown in yellow.

In order to determine the ligand binding pockets of the FGFR2 kinase domain, we utilized the POCASA web server. Predicted binding pockets are shown in Figure 2 and Table 2. The pocket ranked number 1 by POCASA had a volume of 293 Å and a volume depth (VD) of 766. Pockets 2 and 3 had volumes of 92 Å and 41 Å and VD of 230 and 91, respectively. Additionally, the largest binding pocket appeared within the ATP binding cleft. The diversity of pocket volumes and VD indicate that this receptor can bind to a variety of ligands, predictive of effective binding between the receptor and peptides and conjugates [51].

### 3.3. Molecular Docking Studies

Receptor–ligand molecular docking studies play a critical role in predicting the binding interactions and binding affinities of designed drugs or drug conjugates with a specific target receptor [52]. We utilized molecular docking to determine the binding affinities of each of the peptides and peptide–DOX conjugates with the FGFR2 kinase domain. Binding affinities obtained from Autodock Vina of the molecules of interest with the receptor are shown in Table 3.

Overall, L-pep showed the highest binding affinity at −8.2 kcal/mol, followed by Trimer-pep at −6.7 kcal/mol and A-pep at −6.0 kcal/mol. Interestingly, the DOX conjugates each showed increased binding affinities compared to the neat peptides. L-pep-(DOX)_2_ showed the highest binding affinity at −9.6 kcal/mol, while Trimer-pep-(DOX)_2_ displayed a binding affinity of −8.3 kcal/mol. Additionally, compared to DOX alone (−8.4 kcal/mol), both (A-pep)-DOX and L-pep-(DOX)_2_ showed increased binding affinities. These results indicate that while the peptides interact and bind to the FGFR2 kinase domain, conjugation with DOX further enhances the binding interactions.

### 3.4. Protein–Ligand Interaction Profiler (PLIP) Analysis

**Figure 3:**
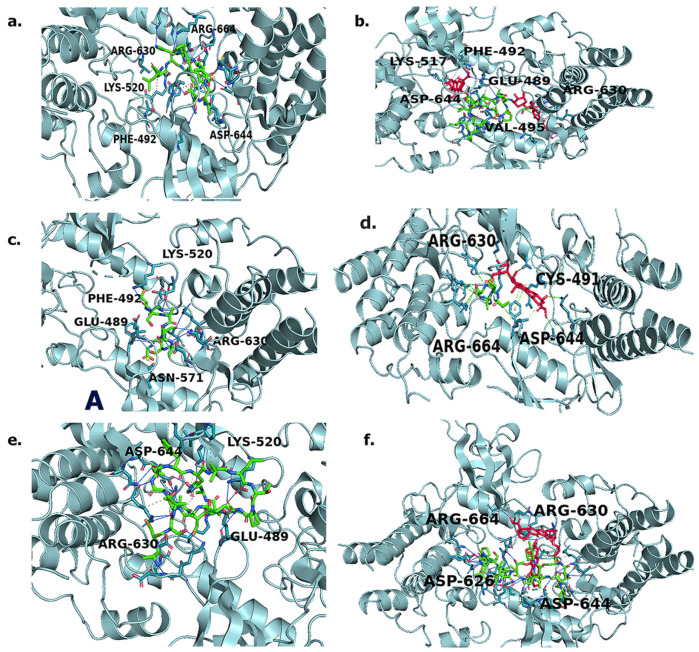
Results of molecular docking studies with FGFR2 kinase domain with peptides and drug conjugates (**a**) L-pep; (**b**) L-pep-(DOX)2; (**c**) A-pep; (**d**) A-pep-DOX; (**e**) Trimer-pep; (**f**) Trimer-pep-(DOX)_2_. Ligands are color coded as follows. Green = peptide component; Red = DOX component.

To gain further insight into the binding interactions, the protein–ligand profiler webserver was utilized to determine the specific residues involved, and those results are shown in Figure 3 and Supplementary Tables S1–S6. L-pep, docking with the FGFR2 kinase domain (Figure 3a) displayed twelve hydrophobic interactions and eleven hydrogen bonds, from the A and B chains of the receptor and one salt bridge, with ARG630 residue. Interestingly, CYS491 (from the glycine-rich loop) and LYS520, as well as notable residues including ASP644, and ARG664 (which are part of the activation loop), were involved in both hydrophobic interactions and hydrogen bond interactions. This is particularly striking because in a recent study involving second-generation FGFR inhibitors, the CYS491 residue was specifically noted as being a necessary binding site in order for the inhibitors to target the FGFR2 kinase domain [53]. Thus, binding interactions with this residue is encouraging and provides support for the ability to target FGFR2. Interestingly, when L-pep-(DOX)_2_ conjugate was docked with the receptor (Figure 3b), we observed an increase in the number of hydrogen bonds to nineteen, while the number of hydrophobic interactions was found to be nine. The increase in the number of hydrogen bonds could be attributed to the interactions with the DOX component. Most notable, however, was that the salt bridge with ARG630 was no longer seen, while interactions with PHE492 and CYS491 from the Glycine rich P-loop were seen. A new hydrophobic interaction with LEU633 within the catalytic region of the FGFR2 kinase domain was seen, in addition to ARG630. Interestingly, many of the new interactions (compared to the peptide alone) occurred within the αC-helix region residues, including ASP521, ASP522, THR524, GLU534 and ASP527, as well as with ALA567 and ASN571 from the hinge region [54]. A hydrogen bond is also seen with ASP644 of the DFG motif. Thus, compared to the neat L-pep the corresponding DOX conjugate shows more interactions, particularly encompassing the A- loop, G-rich loop and the hinge region residues.

The ACSAG peptide sequence (A-pep) displayed a relatively smaller number of interactions compared to L-pep, which is expected, given that it is a shorter sequence (Figure 3c). It showed only four hydrophobic interactions and five hydrogen bonds. Similar to L-pep, the A-pep also showed hydrophobic interactions with several residues in the P-loop region, including LEU487, CYS491, PHE492 and VAL495 [55], while hydrogen bonds occurred with the hinge region residue ASN571, the αC-helix region residue LYS517 and with ASP644 of the DFG motif of the A-loop. The ACSAG-DOX (Figure 3d) conjugate, on the other hand, showed an increased number of hydrogen bonds (thirteen), while there was a decrease in hydrophobic interactions (three). While some interactions with the P-loop and αC-helix residues remained, additional hydrogen bonds also occurred with two activation loop residues ASP644 and ARG664, as well as with the catalytic loop residues ARG630 and ASN631. A new π-stacking interaction was seen with Phe 492, due to stacking with the aromatic residues of DOX. These results indicate that A-pep-DOX conjugate had significantly higher interactions compared to A-pep. Furthermore, similar to the L-pep-(DOX)_2_ conjugate, the A-pep-DOX conjugate continued to interact with the activation loop. In particular, the ARG664 residue interaction is significant because it is known to play a critical role in ATP binding [56].

The highest number of binding interactions amongst the peptides was observed for the Trimer-pep (Figure 3e) designed by combining the peptides described earlier. Overall, eighteen hydrogen bonds and thirteen hydrophobic interactions were seen. In addition, a salt bridge with LYS520 was also seen. This peptide bound to the FGFR2 kinase domain making key contacts with several regions from the kinase domain including the P-loop, hinge region, αC-helix, as well as with the activation loop residues (hydrogen bonds with ASP644 and ARG664 and hydrophobic interactions with ALA643 and LEU647) [57,58]. The hydrogen bond interactions were further enhanced when the Trimer-pep was conjugated with DOX (Figure 3f), displaying thirty hydrogen bond interactions, while the number of hydrophobic interactions was found to be seven.

It is, however, worth noting that a new hydrogen bond interaction with ASP626 is seen, while ASP644 is involved in a hydrophobic interaction with the conjugate instead of a hydrogen bond. Most other interactions were similar to those observed for the trimer sequence. In addition, instead of LYS517, LYS520 was involved in both the salt bridge and H-bonding for the conjugate. As a control, we also examined the binding interactions with neat DOX (Supplementary Figure S1 and Table S7). It displayed only three hydrophobic interactions and seven hydrogen bonds. Specifically, CYS491 and PHE492 were involved in both hydrogen bonds and hydrophobic interactions. In addition, GLY493, LYS517, ASP527 and ASP644 were involved in hydrogen bonding. Overall, these results indicated that the peptide–DOX conjugates, as well as the neat peptides, displayed higher numbers of key interactions within the FGFR2 kinase domain compared to DOX, thus accounting for overall higher binding affinities. Similar results were seen when DOX was conjugated to a peptidiomimetic, Arg-aminonaphthylpropionic acid-Phe for targeting the HER2 receptor, which is also a tyrosine kinase receptor [59].

### 3.5. Molecular Dynamics Simulations

**Figure 4:**
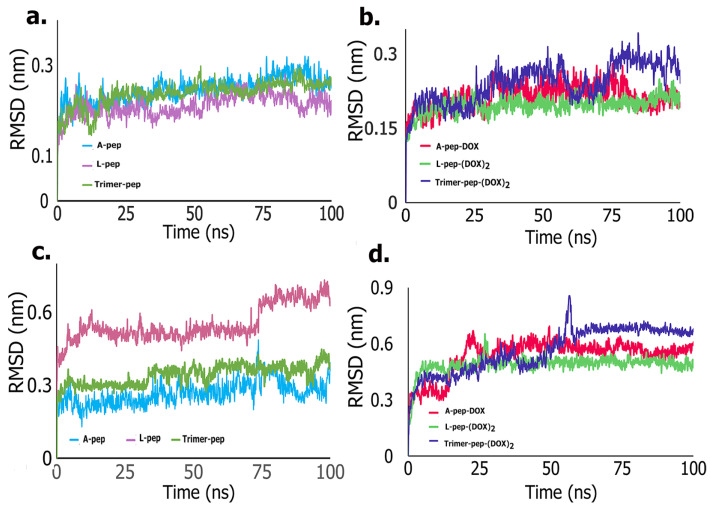
Root mean square deviation (RMSD) plots for complexes of the peptides and peptide–DOX conjugates with FGFR2 kinase domain. (**a**) Cα RMSD plots with the neat peptides; (**b**) Cα RMSD plots with the neat peptide–DOX conjugates; (**c**) RMSD plots of protein–ligand complex with the neat peptides; (**d**) RMSD plots of protein–ligand complex with the DOX conjugates of the peptides.

**Figure 5:**
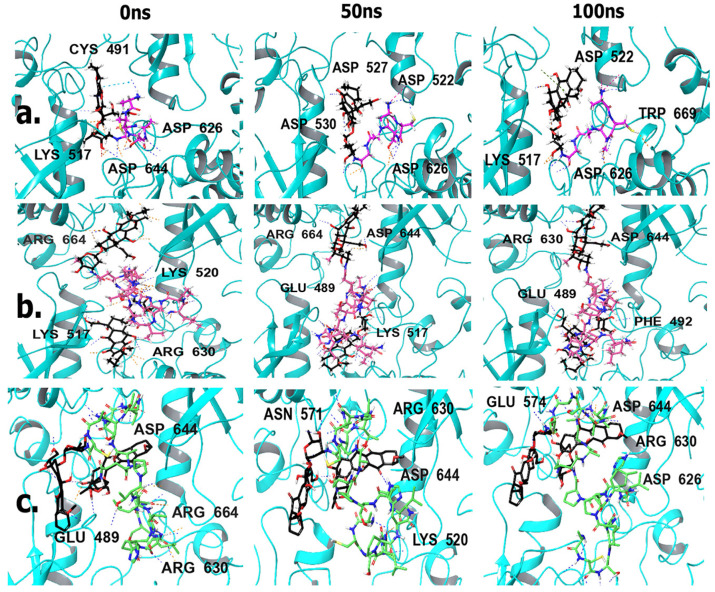
Trajectory images showing interactions of peptide–DOX conjugates with FGFR2 over 100 ns simulations. (**a**) A-pep-DOX (**b**) L-pep-(DOX)_2_ (**c**) Trimer-pep-(DOX)_2_. The black portion of the ligand indicates DOX, and green or pink indicates peptide segments.

**Figure 6:**
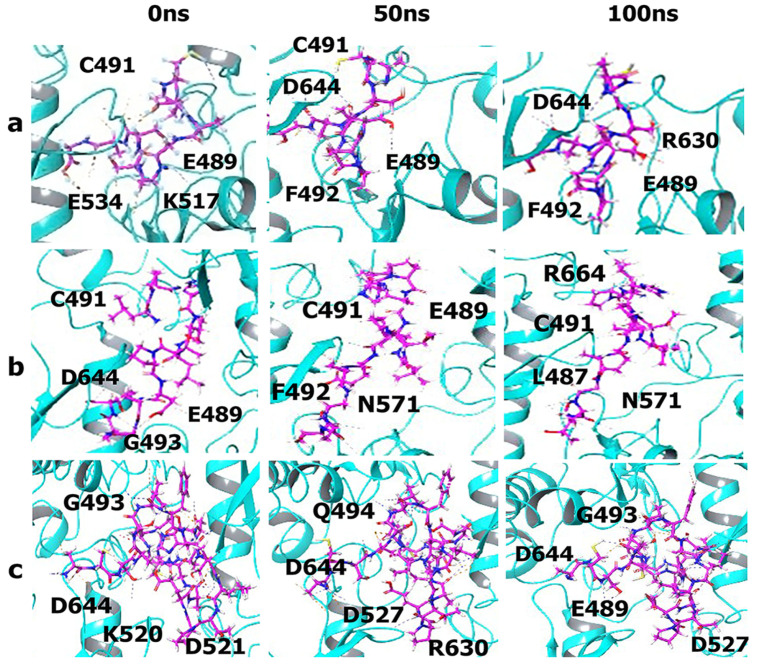
Trajectory images showing interactions of the three peptides with FGFR2 over 100 ns simulations. (**a**) A-pep; (**b**) L-pep; (**c**) Trimer-pep at 0 ns, 50 ns and 100 ns (from left to right).

To further elucidate the binding interactions, and compare the stabilities of the complexes of the peptides and the conjugates with the FGFR2 kinase domain, we conducted molecular dynamics (MD) simulations. As can be seen in Figure 4, the Cα root mean square deviation (RMSD) values seen upon binding to the peptides and the peptide–DOX conjugates were similar (0.25 nm to 0.32 nm). After an initial increase in the first two nanoseconds, there were very few deviations seen in the RMSD values for the rest of the simulation (Figure 4a,b). In comparison, there was a slight increase in the receptor–ligand RMSD values upon binding to the three peptides (Figure 4c). The lowest RMSD value was seen for A-pep, followed by the Trimer-pep. The L-pep, however, showed an increase in RMSD value to 0.5 nm, which remained stable up to 70 ns, after which the RMSD value increased to 0.62 nm. These changes were within 0.4 nm of the Cα values, and were therefore considered stable. Furthermore, these results indicate that A-pep and the Trimer-pep formed more stable complexes compared to L-pep. Interestingly, amongst the conjugates (Figure 4d), however, the A-pep-DOX conjugate showed a gradual increase initially to 0.6 nm; however, after 25 ns, it remained relatively stable at 0.45 nm, likely due to initial conformational changes. Comparatively, the L-pep-(DOX)_2_ conjugate showed significantly lower RMSD values, where the values remained within 0.4 nm and 0.5 nm throughout the simulation. In the case of the Trimer-pep-(Dox)_2_, however, the RMSD value increased to 0.75 nm between 60 and 65 ns, then decreased to 0.65 nm, and showed no significant changes after 70 ns. These results indicate that the Trimer-pep-(Dox)_2_ may be undergoing more conformational changes within the receptor; however, all of the ligands remained firmly attached throughout the simulation. Furthermore, the results correlate strongly with the trajectory images, which show binding interactions for the peptide–DOX conjugates (Figure 5) and the peptides within the FGFR2 kinase domain (Figure 6). In particular, the Trimer pep-(DOX)_2_ conjugate appears to be shifting within the binding pocket, where initial interactions with residues such as GLU489 from the P-loop region are seen at 0 ns, while new interactions with LYS520 near the αC helix region are seen at 50 ns. Interestingly the ASP644, ASP664 and ARG630 interactions are seen throughout the simulation, implying their role in binding. A-pep-DOX conjugate, on the other hand, remained attached to ASP626 throughout the simulation, while interactions with ARG630 appeared after 25 ns and remained throughout the rest of the simulation. However, initial interactions with the P-loop residue CYS491 and the activation loop residue ASP644 were not seen after 50 ns of the simulation. Instead, new interactions with TRP669 are seen at the end of the simulation. For the L-pep-(DOX)_2_ conjugate, however, the conjugate continues to interact with the P-loop residue GLU489 after 50 ns of the simulation, though the initial interaction with LYS517 is lost after 50 ns. However, it is promising that the activation loop residue ASP644 and catalytic loop residue ARG630 are seen at the end of the simulation. In contrast, the A-pep, being only a pentapeptide, appears to become more compact within the binding pocket and remains firmly attached to the P-loop residue CYS491 for the first 50 ns, along with GLU534 and LYS517. By the end of the simulation, it is seen to interact with PHE492 and GLU489 along with ARG630 and ASP644. The L-pep appeared to be well spread out within the binding pocket throughout the simulation, though subtle movement is seen. A majority of the interactions are seen with P-loop residues, though, after 50 ns, the peptide also interacts with the hinge region residue ASN571. However, ASP644, which is seen to initially interact with the L-pep, is no longer seen; instead, a new interaction with ARG664 is seen at the end of the simulation. The Trimer-pep also remains firmly attached within the binding pocket, though a slight change in interactions is observed. By the end of the simulation, residues from the activation loop, αC-helix and P-loop are seen to interact with the trimer peptide. Thus, the trajectory analyses further revealed the involvement of many residues first identified in docking studies. Specifically, residues such as ASP644, ARG664, ARG630 or CYS491 were involved in binding with the peptides or drug conjugate. Additionally, ASN571 was also observed to be involved in binding with L-pep. ASN571 is of interest because has also been implicated in binding with FGFR2 inhibitors [60]. The involvement of each of these residues provides further support for their potential as FGFR2 targeting agents, particularly because these interactions were retained upon DOX conjugation.

**Figure 7:**
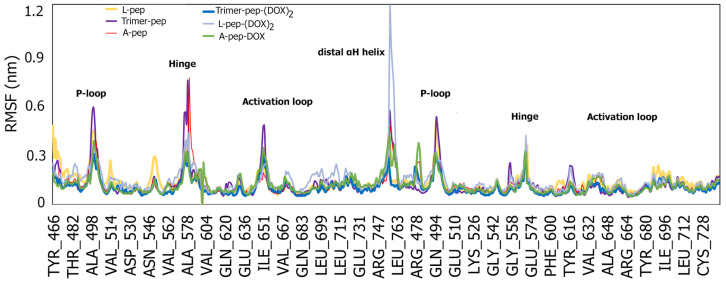
Root mean square fluctuation plots of the FGFR2 kinase domain upon interations with A-pep, L-pep, Trimer-pep and their respective conjugates.

The root mean square fluctuation results (Figure 7) displayed peaks in similar regions for all peptides and DOX conjugates. However, the highest fluctuation was seen for the L-pep-(DOX)_2_ conjugate at LEU757 in the distal alpha-H-helix region. Other prominent residues showing relatively higher fluctuations included VAL495, ALA500, ARG580 and ASN652, implying higher flexibility of those regions (P-loop; hinge region and A-loop residues) upon binding to the peptides or the peptide–drug conjugates. Overall, these results further demonstrate that the peptides and drug conjugates form critical interactions with the kinase domain of FGFR2 receptor.

#### MM-GBSA Analysis

The molecular mechanics energies combined with generalized Born and surface area continuum solvation (MM-GBSA) calculations allow for the estimation of the free energy of binding for molecular dynamics simulations of receptor–ligand complexes [61]. The results obtained for peptides and their respective DOX conjugates are shown in Table 4. The results followed similar trends to those of the molecular docking studies, in that upon DOX conjugation, the overall binding energies were enhanced. The highest average ΔG of binding energy among the peptides was observed for the A-pep, with a ΔG of −135.5 kcal/mol, while among the conjugates, L-pep-(DOX)_2_ showed the highest average ΔG value, at −157.3 kcal/mol. The lowest overall binding was seen for L-pep at −78.8 kcal/mol. Furthermore, these results indicate that Van der Waals forces largely contributed to the interactions in all cases.

### 3.6. Laboratory Studies

#### 3.6.1. SPR Analysis

In order to further validate the binding interactions of the peptides with the FGFR2 receptor, we conducted SPR analysis. As shown in Table 5, among the three peptides studied, the lowest K_D_ value (highest binding affinity) was seen for the Trimer-pep. The A-pep showed the next-highest binding affinity, while the L-pep sequence showed the highest K_D_ value (least binding). Overall, these results corroborate with the results obtained from MD simulation studies, indicating that each of the peptides successfully interacted with the receptor and that the Trimer-pep and A-pep demonstrated stronger binding. This is expected, given the presence of the charged residues along with hydrophilic residues Thr and Ser, as well as hydrophobic residues including Leu, Ala and Val, which can interact with the binding pocket residues of the FGFR2 kinase domain. We also compared our results with that of a known FGFR kinase inhibitor (CAS 192705-79-6), a small-molecule inhibitor which showed the lowest K_D_ value. Overall, these results indicate that though the binding affinity is stronger for the control, the peptides designed also showed binding to the kinase domain of FGFR2.

#### 3.6.2. FTIR Spectroscopy

**Figure 8:**
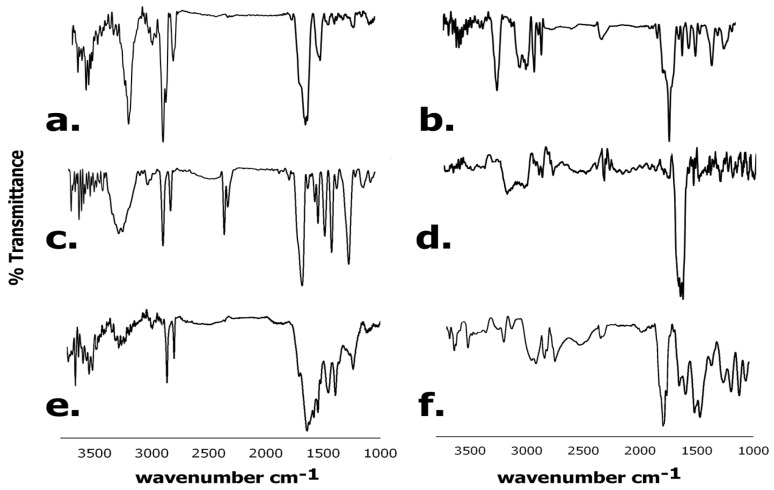
Comparison of FTIR spectra and neat peptides and DOX conjugated peptides. (**a**) A-pep; (**b**) A-pep-DOX; (**c**) Trimer-pep; (**d**) Trimer-pep-(DOX)_2_; (**e**) L-pep; (**f**) L-pep-(DOX)_2_.

Upon confirmation that the peptides successfully attached to the receptor, we conjugated the peptides with the chemotherapeutic drug DOX to enhance their efficacy for tumor targeting. The formation of the DOX conjugates was also confirmed by FTIR spectroscopy. Results obtained are shown in Figure 8. As can be seen, for the A-pep–DOX conjugate, the amide I peaks are seen at 1653 cm^−1^ and at 1633 cm^−1^, while the neat peptide displayed a peak at 1638 cm^−1^. Additionally, the C-O peak showed a shift from 1231 cm^−1^ to 1213 cm^−1^ and the amide II peak was shifted to 1518 cm^−1^ for the DOX conjugate compared to 1515 cm^−1^ and a short peak at 1547 cm^−1^. Furthermore, the carboxyl peak at 3580 cm^−1^ seen for the peptide was significantly diminished due to conjugation with DOX; however, a short peak appears in the region at 3521 cm^−1^ due to the phenolic hydroxyl groups of DOX. Both A-pep and its DOX conjugate show strong peaks due to -CH stretching in between 2915 cm^−1^ and 2970 cm^−1^; as a result of -CH stretching, the -NH stretching peak is observed at 3264 cm^−1^ for the A-pep, which shifts to 3281 cm^−1^ for the A-pep DOX conjugate. For the Trimer-pep, the amide I peak was observed at 1652 cm^−1^. However, upon conjugation with DOX, a shift was observed to 1660 cm^−1^ in addition to the appearance of a shoulder at 1681 cm^−1^. These peaks are typical of the presence of DOX [62]. We also observed a shift in the C-O peak from 1231 cm^−1^ (for the neat Trimer-pep) to 1215 cm^−1^ for the corresponding DOX conjugate. Furthermore, the amide II peaks were shifted to 1565 cm^−1^ and to 1548 cm^−1^ for the conjugate compared to 1539 cm^−1^ and 1513 cm^−1^ for the peptide. Similarly, For the L-pep sequence, peaks were seen at 1643 cm^−1^ with a shoulder at 1619 cm^−1^ for amide I, while amide II peaks were seen at 1540 cm^−1^ and at 1576 cm^−1^.

Additional sharp peaks were seen at 1451 cm^−1^ and 1394 cm^−1^ for -OH bending vibrations, and at 1234 cm^−1^ for C-O stretching. However, for the corresponding DOX conjugate, the amide I peaks were seen at 1667 cm^−1^ and at 1655 cm^−1^ with a shoulder at 1627 cm^−1^. The amide II peak was shifted to 1524 cm^−1^, while the C-O stretching peak was shifted to 1258 cm^−1^. Both L-pep and Trimer-pep showed changes in the 3500–3600 cm^−1^ region upon conjugation with DOX. Similar shifts were observed when the tumor-targeting peptide conjugated with DOX pHLIP-SS-DOX was prepared to target MCF-7 tumor cells [63]. These results further confirm the conjugation of DOX with the peptides.

#### 3.6.3. DSC Analysis

**Figure 9:**
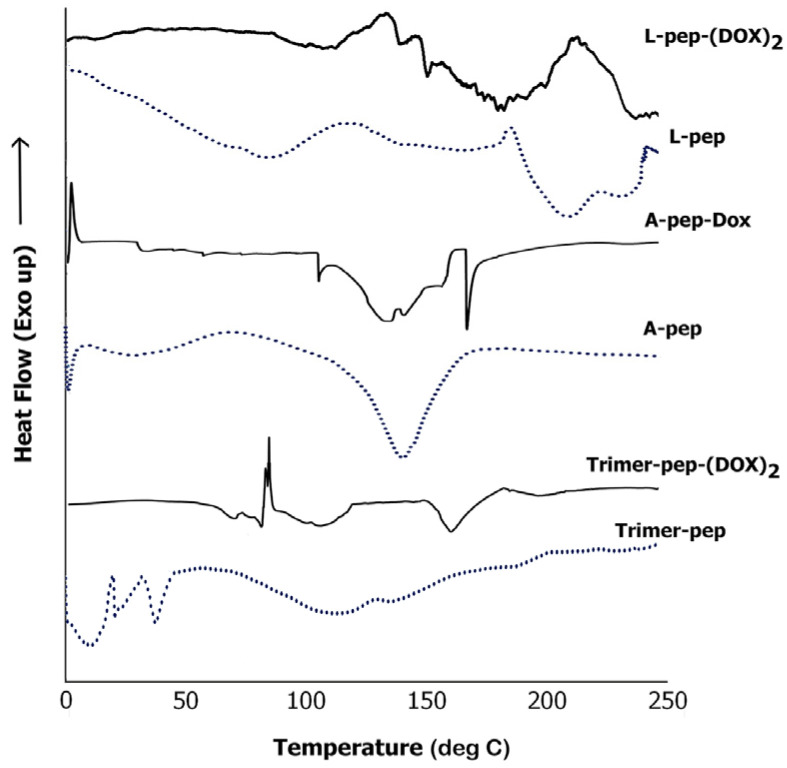
Comparison of DSC thermograms of A-pep; L-pep and Trimer-pep and their respective DOX conjugates.

We conducted DSC analysis of the DOX conjugates and that of the neat peptides to examine the phase changes that occurred, and in order to investigate the thermal behavior of the peptides and conjugates [64]. As can be seen in Figure 9, each of the unbound peptides exhibited a broad endothermic peak characteristic of peptide unfolding, primarily due to moisture loss and thermal dehydration [65]. While the L-pep displayed a broad endothermic peak at 79.2 °C, A-pep displayed a sharp endothermic peak at 1.5 °C due to loss of water, followed by a short broad endothermic peak at 42.1 °C, indicating that the L-pep unfolds at a relatively higher temperature compared to A-pep. The Trimer-pep showed two short endothermic peaks at 10.2 °C and at 37.2 °C due to water loss, followed by broad endothermic peaks at 110 °C at 145 °C due to unfolding and thermal melting. Additionally, the L-pep displayed endothermic peaks at 210.3 °C and at 235 °C due to thermal decomposition. In the case of the A-pep, the thermal melting peak was seen at 147.2 °C.

Interestingly, the L-pep-(DOX)_2_ conjugate showed a broad melting peak between 155 °C and 194.6 °C likely due to melting of the peptide and the doxorubicin component of the conjugate. Similar broad endothermic peaks were observed when DOX was conjugated with bovine lactoferrin protein due to the formation of the hybrid peptide–drug conjugate [66]. The melting temperature seen here is lower than pure doxorubicin, as expected, given the fact that it was conjugated to the peptide. In addition, there were two short melting peaks at 133.2 °C, and 144.6 °C as well as a short broad endothermic peak at 105 °C due to unfolding of the peptide and water loss. The A-pep-DOX conjugate showed a crystallization peak at 2.1 °C due to water content. The thermal broad melting peaks for the conjugates appear to be in a similar range as that seen for the neat A-pep. There is an additional melting peak at 165.4 °C, which can be attributed to the DOX component of the conjugate. The Trimer-pep-(DOX)_2_ conjugate displayed a sharp crystallization peak at 86.5 °C, followed by a short broad melting peak due to unfolding of the peptide. An additional endothermic peak was seen 160.5 °C for the conjugate attributed to the DOX component. The Trimer-pep-(DOX)_2_, being the largest of the sequences, may display additional phase changes such as the crystallization peak, not seen in the other two conjugates due to thermal unfolding and reorganization caused by changes in hydrogen bonds and other non-covalent interactions of the conjugate when heated [67]. Furthermore, the presence of multiple transition peaks is indicative that this particular conjugate may undergo stepwise phase transitions.

#### 3.6.4. Cell Studies

As a tumorigenic cell model, we utilized KATO (III) gastric cancer cells. We specifically utilized KATO III gastric tumor cells as they are known to overexpress the FGFR2 receptor [68]. Furthermore, kinase inhibition was found to reduce the growth of those tumor cells. To confirm expression of FGFR2, we conducted ELISA studies. The expression of FGFR2 was confirmed to be 15.2 pg/mL.

**Figure 10:**
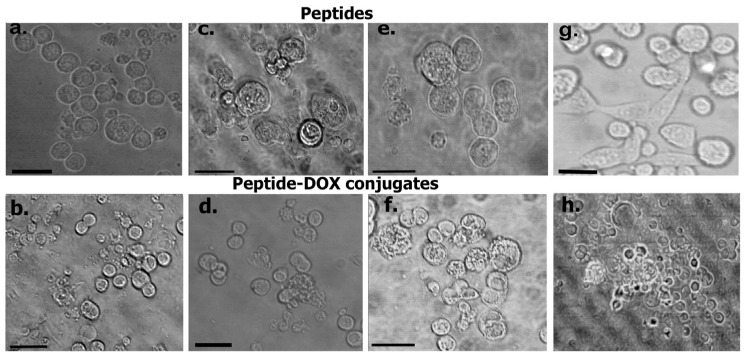
Impact of peptides and DOX conjugates on KATO III cells after treatment for 24 h with (**a**) L-pep; (**b**) L-pep-(DOX)_2_; (**c**) A-pep; (**d**) A-pep-DOX; (**e**) Trimer-pep; (**f**) Trimer-pep-(DOX)_2_; (**g**) Control untreated cells; (**h**) DOX-treated cells. Data shown are those obtained after treatment with 1 µM peptide or conjugate and DOX. (**a**,**b**,**d**,**f**,**g**,**h**) Scale bar = 75 µm; (**c**,**e**) Scale bar = 50 µm.

To investigate the impact of the peptides and the peptide–DOX conjugates on KATO (III) tumor cells, we conducted cytotoxicity studies. The results obtained are shown in Figure 10. As can be seen, the cells treated with the L-pep and its DOX conjugate appeared spherical, though some blebbing was observed upon treatment with the conjugate. In comparison, upon treatment with the A-pep and its DOX conjugate, cells appeared to show loss of morphology and adhesive properties due to cortical remodeling. In particular, the cells treated with A-pep-DOX appeared to bleb and burst, indicating that cells were breaking apart. Treatment with the Trimer pep and its DOX conjugate resulted in partial loss of morphology. These results indicated that the A-peptide and the Trimer-pep and their DOX conjugates caused changes in cell morphology of the KATO (III) cells. The unconjugated neat DOX (1 μM) treated cells also displayed similar morphology as that seen for the trimer-pep-(DOX)_2_ conjugates. In comparison, the control untreated KATO (III) cells were found to show typical adherent morphology with cytoskeletal elongation along with spherical clusters of cells [69].

**Figure 11:**
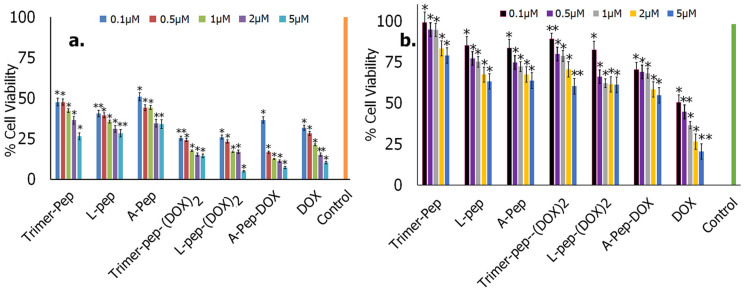
Percent cell viability of cells upon treatment with peptides, DOX peptide conjugates and neat DOX after 24 h of treatment. (**a**) KATO (III) tumor cells; (**b**) Normal lung fibroblast cells. (* *p* < 0.5; ** *p* < 0.005).

To quantify the effects of the cytotoxicity of the peptides and the DOX conjugates, we conducted WST-8 assays. As a control, and to examine specificity toward tumor cells, we also carried out cytotoxicity assays with non-tumor normal lung fibroblast cells. Results obtained after 24 h of treatment are shown in Figure 11. In Figure 10a, the data show that, overall, the cytotoxicity was found to be concentration-dependent, with treatment at higher concentrations demonstrating higher cytotoxicity. As expected, the conjugates demonstrated higher cytotoxicity compared to the peptides; however, A-pep-DOX and L-pep-(DOX)_2_ conjugates demonstrated the highest cytotoxicity (viability of 9% and at 7%, respectively, at the highest concentration). Interestingly, all of the peptides also caused reduction in viability to 52% or below, depending upon the concentration of the peptide used. We then compared the effects on non-cancer cells. As can be seen in Figure 10b, even though there was a marginal reduction in viability, (to 65–70% for the conjugates) and up to 72% upon treatment with the peptides, compared to neat DOX, the viability of the non-cancer cells remained slightly higher (65–75%). These results indicated that the peptides and their DOX conjugates were more potent and targeted the gastric tumor cells more strongly.

#### 3.6.5. Growth of Spheroids

To mimic the cellular complexity of tumors, we grew 3D co-cultured spheroids composed of KATO (III) cells and cancer-associated fibroblasts (CAFs) at a 3:1 ratio at two different densities (2000 cells/well and 4000 cells/well). We specifically selected CAFs for co-culturing because those cells are one of the most copious constituent cells of the tumor microenvironment and are known to play an important role in metastasis through cell–cell interactions and secretion of pro-invasive factors such as extracellular matrix (ECM)-degrading proteases [70]. Several methods have been developed to mimic the tumor microenvironment. For example, previous studies have shown that tumor cells have been encapsulated in PEGDA gels, followed by micropatterning to optimize the growth of tumoroids [71]. In this study, the spheroids were grown in U-bottom low-attachment sphera 3D plates to promote the growth of spheroids [72]. The growth of the spheroids was monitored over time, and the double-stranded DNA content was examined at varying time points. To determine DNA content, the Quant-iT dsDNA assay was conducted. The results obtained after three days and ten days of growth are shown in Table 6. As can be seen, the DNA content showed an increase over time, confirming the growth of spheroids [73].

##### Spheroid Imaging

**Figure 12:**
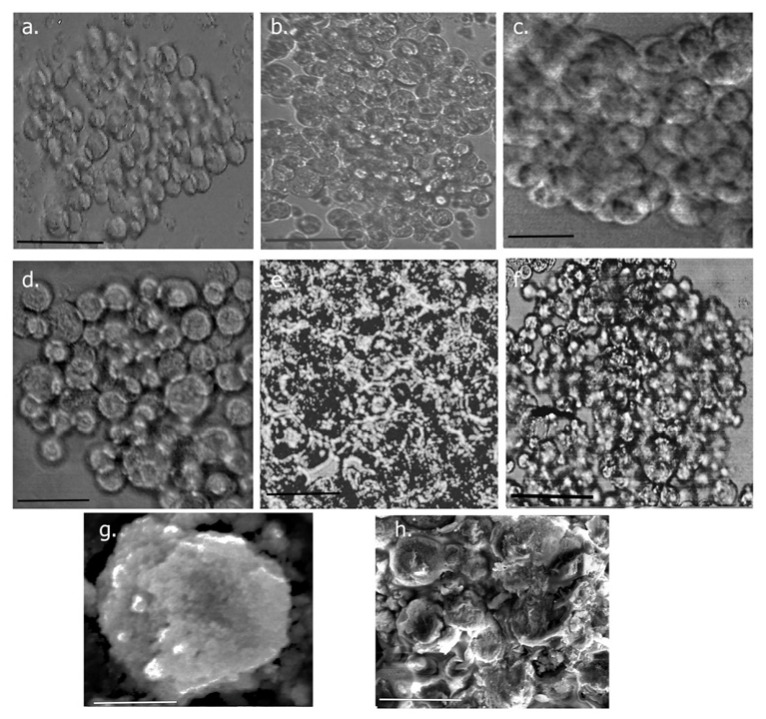
Growth of spheroids over time. (**a**) through (**c**) includes cells grown at a density of 2000 cells/mL; (**d**) through (**f**)indicate spheroids grown at a density of 4000 cells/mL. (**a**,**d**) after 24 h; (**b**,**e**) after 144 h and (**c**,**f**) after 240 h of growth. Scale bar = 80 µm. (**g**) SEM image of a spheroid grown at 2000 cells/well after 12 days of growth. Scale bar = 30 µm; (**h**) SEM image of a spheroid grown at 4000 cells/well after 12 days of growth. Scale bar = 20 µm.

To further examine the growth of spheroids, the co-cultures were grown at two different cell-seeding concentrations in micro-wells, and the diameter, solidity and shape were investigated. Cells grown at 2000 cells/microwell and 4000 cells/microwell are shown in Figure 12 at time periods of 24 h, 144 h and 240 h of growth. At 24 h, cells appeared to come together to form cell aggregates in each of the wells, and individual cells could be easily identifiable. Over time, cellular aggregates appeared to fuse together, forming multilayered cellular structures which then formed solid spheroids. Furthermore, in addition to KATO III cells, CAFs are also seen throughout the spheroids, and exhibited an irregular elongated and flattened morphology, which is typical of tumor-associated fibroblasts [74]. This was particularly more pronounced for the 4000-cell wells. At day 10, we did not see a significant change in the size. The results showed darkening in some areas, indicative of the formation of necrotic cells, possibly due to low oxygen conditions [75]. We also conducted SEM imaging to examine the morphology of the tumoroids. As can be seen, multilayered tumoroids were observed. Additionally, the cells grown at higher concentrations formed more spherical morphologies.

### 3.7. Permeation of Peptides and DOX Conjugates into 3D Spheroids

**Figure 13:**
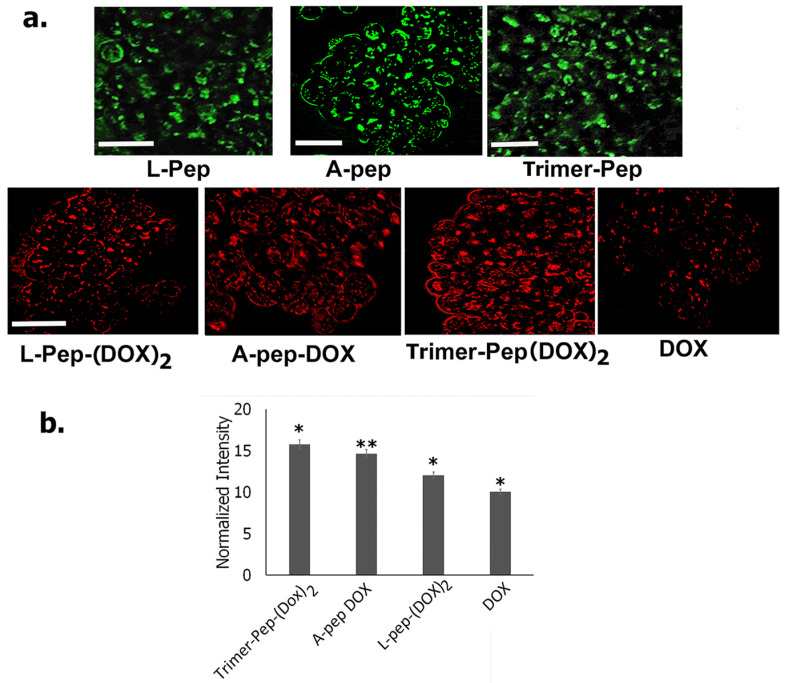
(**a**) Top row: Fluorescence images of carboxyfluorescein- tagged peptides after incubation with spheroids grown for 6 days at a density of 2000 cells/mL. Bottom row: Fluorescence images of DOX-conjugated peptides after incubation with spheroids grown for 6 days at a density of 2000 cells/mL. (**b**) Semiquantitative analysis of the mean fluorescence intensity in spheroids treated with DOX-peptide conjugates. Data represented as mean from 3 spheroids for each treated conjugate. Statistical Analysis was performed using student *t*-tests. * *p* < 0.05, ** *p* < 0.01.

It is well known that constrained interstitial penetration may impede the diffusion of drugs into tumors, thus affecting efficacy [76]. To examine if the peptides were successfully able to penetrate into the spheroids, each of the peptides was tagged with carboxy fluorescein and incubated with the spheroids for 24 h. Their entry within the spheroids was examined through fluorescence imaging. In the case of the conjugates, because DOX is inherently fluorescent, fluorescence images were taken without tagging. The results obtained are shown in Figure 13. As can be seen (Figure 13a), each of the peptides were found to permeate into the spheroids. However, in the case of the A-pep and Trimer-pep, there appeared to be higher internalization as is evident by the appearance of higher fluorescence near the core of the spheroids. For the L-pep, relatively higher fluorescence was observed in the outlayers compared to the core of the spheroids. In the case of the DOX-peptide conjugates, relatively higher penetration was observed for the trimer-pep-(DOX)_2_ conjugates. In comparison, in the case of neat DOX, higher fluorescence was observed toward the edges, indicating higher accumulation of the drug in those regions, although marginal internalization was seen. In the case of the trimer-pep-(DOX)_2_ and the A-pep-DOX conjugates, higher fluorescence was observed, while comparatively lower intensity of fluorescence was observed for the L-pep-(DOX)_2_ conjugates. To further quantify these results, we performed Image J analysis. The plot profiles obtained from the edge of the spheroid to the center were measured. On average, five measurements were carried out for images of each of the spheroids that were incubated with DOX-conjugated peptides, as well as with neat DOX. The fluoresence intensity from the periphery to the center of the spheroid was measured. Results are shown in 13b which indicate the mean normalized intensities obtained. The results confirm that the trimer-pep (DOX)_2_-conjugated peptides and A-pep-DOX are able to penetrate deeper into the tumoroids and are not accumulating simply in the periphery. Thus, overall, the peptides may be utilized as carriers for drugs into the spheroids. While these results are promising, it is to be noted that the complexity associated with the heterogenous nature of tumors in vivo is vast. Since there is no vasculature associated with the spheroids developed in this work, the current system may not be reflective of tumoroids in vivo. However, it serves as a model that can be utilized to study the binding interactions of constructs with specific overexpressed receptors of particular cell lines. This study, in particular, demonstrates a semi-quantitative analysis of mean fluorescence intensity of DOX and DOX-conjugated peptides within the spheroids. Further studies will be necessary to examine the pathways involved in internalization and trans-tissue transport in order to develop therapeutics.

### 3.8. Immunofluorescence Studies

**Figure 14:**
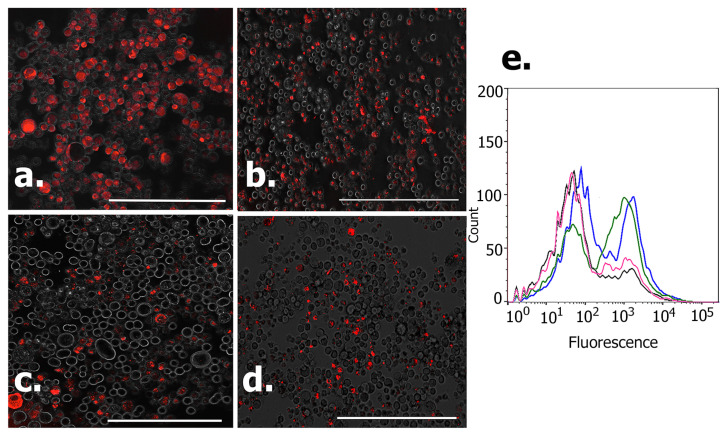
Confocal images of FGFR2 antibody (Alexa Fluor 647)-tagged cells, (**a**) Control untreated KATO (III) cells; Cells treated with (**b**) Trimer-pep; (**c**) A-pep; (**d**) L-pep and then attached to antibodies. (**e**) shows the flow cytometry analysis of the antibody-tagged cells. Blue indicates control cells; pink = L-pep-treated; black = A-pep-treated; green = trimer-pep-treated.

To further assess the impact of the specific peptides and their ability to bind to FGFR2 receptors of KATO (III) cells, we conducted immunofluorescence studies and FACS analysis. Confluent cells were labeled with Alexa Fluor-647-tagged FGFR2 antibodies before and after treatment with each of the peptides. The results are shown in Figure 14. The confocal images of the antibody-labeled cells before and after treatment with peptides are seen in Figure 14a–d. As can be seen, the control cells show higher binding with the antibody, as indicated by higher fluorescence. However, comparatively, lesser numbers of cells appear to bind to the antibody upon pre-treatment with the peptides. Thus, these results further confirm that the peptides are likely to bind to the FGFR2 receptors overexpressed by KATO (III) cells. To further quantify these results, FACS analysis was carried out. As shown in Figure 14e, two distinct populations of cells are seen. This is expected, given that KATO (III) cells are known to demonstrate a stem-cell like side population of cells. [77]. The results demonstrate that after treatment, there was a shift observed to the left for the cells treated with the peptides compared to the control, which showed higher fluorescence due to higher binding with the antibodies. These results further confirm that FGFR2 antibody binding is lower in the presence of the peptides.

## 4. Conclusions and Future Directions

In this work, the ability of two bioactive naturally derived peptide sequences, ACSAG (A-pep) and LPHVLTPEAGAT (L-pep), as well as a newly designed fusion peptide ACSAG-LPHVLTPEAGAT-GASCA (Trimer-pep), to bind to the kinase domain of the FGFR2 receptor was investigated for the first time. Furthermore, each of these peptides was conjugated with the chemotherapeutic drug doxorubicin. The ability of these peptides and the drug conjugates to bind to the FGFR2 receptor kinase domain was studied computationally as well as through laboratory studies. The FGFR2 receptor, which is overexpressed in gastric tumor cells, is a prime target due to its known amplification in gastric cancers that leads to poor prognosis. Molecular docking and MD simulations revealed that each of these peptides successfully bound to the kinase domain of the FGFR2 receptor. MMGBSA studies revealed that among the peptides, the Trimer-pep and A-pep showed stronger binding compared to L-pep. While among the conjugates, A-pep-DOX and L-pep-(DOX)_2_ demonstrated higher binding energies. MD simulations and docking studies revealed that the peptides and their drug-conjugates showed key interactions with the glycine-rich loop (CYS491 and GLU489) and the activation loop residues of the kinase domain (ASP664 and ASP644), and, in some cases, hinge region residues were also involved. Furthermore, the binding of the peptides with the FGFR2 receptor was validated using surface plasmon resonance studies. The peptides were each conjugated to doxorubicin to enhance their efficacies in targeting FGFR2 overexpressed tumor cells. In general, the peptides and their =DOX conjugates were found to show higher cytoxicity toward the tumor cells compared to normal cells, implying a degree of specificity. Three-dimensional spheroids with co-cultures of CAFs and KATO (III) cells were grown, and our results showed that the peptides and DOX conjugates were able to penetrate the 3D tumoroids. Overall, this study shows the potential development of new peptides and peptide–drug conjugates that may possibly target overexpressed FGFR2 receptor kinase domain in tumor cells and may be considered for the development of future therapeutics. However, to further examine the impact on tumoroid activities, further in vivo studies will be necessary, which is beyond the scope of this manuscript. Future directions may involve investigating further mechanistic details into the activity of the peptides and the peptide–drug conjugates, and examining the expression of various cytokines upon treatment of the turmoroids with these constructs. Additional avenues we intend to pursue involve investigating binding interactions of the peptides and the peptide–drug conjugates with mutant forms of FGFR2 which are known to cause drug resistance.

## Figures and Tables

**Table 1:** Anti-CP 2.0 results for peptides.

Peptide	SVM Score	Anti-CP	Hydrophobicity	Hydropathicity	Hydrophilicity	pI
A-pep	1.11	Yes	0.09	0.98	−0.34	5.85
L-pep	0.68	Yes	0.06	0.31	−0.37	5.25
Trimer-pep	0.69	Yes	0.07	0.61	−0.35	5.25

**Table 2:** POCASA results showing pocket indices, volumes and VD values for the kinase domain of FGFR2.

Rank	Index	Volume	VD Value
1	86	293	766
2	95	92	230
3	293	41	91
4	307	25	71
5	312	23	56

**Table 3:** Binding affinity values in kcal/mol for each peptide and DOX conjugate with FGFR2 from docking studies.

Peptide/Conjugate/Drug	Binding Affinity (kcal/mol)
ACSAGLPHVLTPEAGATGASCA (Trimer-pep)	−6.7
LPHVLTPEAGAT (L-pep)	−8.2
ACSAG (A-pep)	−6.0
Trimer-pep-(DOX)_2_	−8.3
L-pep-(DOX)_2_	−9.6
(A-pep)-DOX	−8.6
DOX	−8.4

**Table 4:** MMGBSA Analysis.

Peptide/Conjugate	ΔG Bind (kcal/mol)	ΔG Coulomb (kcal/mol)	ΔG Hydrogen Bonds (kcal/mol)	ΔG Lipophilic (kcal/mol)	ΔG Solvation (kcal/mol)	ΔG VdW (kcal/mol)
Trimer-pep	−111.9	−60.3	−8.4	−27.3	92.3	−125.1
L-pep	−78.8	−43.8	−4.9	−17.8	59.9	−77.2
A-pep	−135.5	92.8	−7.8	−27.5	−88.2	−111.5
Trimer-pep-(DOX)_2_	−97.6	−60.3	−8.8	−16.4	95.2	−119.0
L-pep-(DOX)_2_	−157.3	112.6	−8.6	−33.6	−110.7	−124.1
A-pep-DOX	−153.9	104.7	−8.5	−32.7	−103.8	−119.8

**Table 5:** Determination of K_D_ values from SPR Analysis.

Peptide	K_D_ Values (M)
A-pep (ACSAG)	(14.05 ± 2.8) × 10^−6^
L-pep (LPHVLTPEAGAT)	(22.61 ± 0.8) × 10^−6^
Trimer-pep (ACSAGLPHVLTPEAGATGASCA)	(7.53 ± 1.2) × 10^−6^
FGF Receptor Tyrosine Kinase Inhibitor—CAS 192705-79-6 (CONTROL)	(1.9 ± 3.2) × 10^−7^

**Table 6:** DNA content of spheroids over time.

Spheroids	DNA Content at 72 h (ng/mL)	DNA Content at 240 h (ng/mL)
Spheroids grown at 4000 cells/well	246	492.6
Spheroids grown at 2000 cells/well	164	330.2

## Data Availability

The original contributions presented in the study are included in the article and in Supplementary Materials.

## Supplementary Materials

The following supporting information can be downloaded at: https://www.mdpi.com/article/10.3390/biomimetics9090515/s1, Tables S1–S6: which show the protein–ligand interactions for the peptides and their corresponding doxorubicin conjugates; Table S7 and Figure S1 represent binding interactions and molecular docking results of DOX (doxorubicin) with the FGFR2 kinase domain. DOX is represented in red.
