# Supplementary material for: Developing New Peptides and Peptide–Drug Conjugates for Targeting the FGFR2 Receptor-Expressing Tumor Cells and 3D Spheroids
Source: Biomimetics (Basel). 2024 Aug 27;9(9):515. doi: 10.3390/biomimetics9090515 (PMC11429203; doi:10.3390/biomimetics9090515)
Supplement: Supplementary file 1 [file biomimetics-09-00515-s001.zip › biomimetics-3096303-supplementary.pdf]

Developing New Peptides and Peptide-Drug Conjugates for Targeting the FGFR2 Receptor Expressing Tumor Cells and 3D Spheroids

Mary A. Biggs, Amrita Das, Beatriz G. Goncalves, Molly E. Murray, Sophia A. Frantzeskos, Hannah L. Hunt, Chau Ahn N. Phan and Ipsita A. Banerjee *

Department of Chemistry and Biochemistry, Fordham University, 441 East Fordham Road, Bronx, New York 10458, USA

Table S1. Binding interactions of LPHVLTPEAGAT (L-Pep) with FGFR2 Kinase domain (A and B are indicative of the A and B chains involved).

Hydrophobic Interactions		H-Bond Interactions		Salt Bridges		π-stacking	
Residue	Distance (Å)	Residue	Distance (Å)	Residue	Distance (Å)	Residue	Distance (Å)
Cys 491B	3.69	Cys 491B	2.18	Arg 630B	4.25		
Phe 492B	3.90	Gly 493A	2.44				
Phe 492B	3.73	Lys 517A	3.26				
Lys 520A	3.78	Lys 517B	2.21				
Thr 524A	3.62	Lys 520A	3.21				
Thr 524B	3.87	Asp 522A	2.95				
Asp 644B	3.54	Asp 527A	2.43				
Leu 647A	3.49	Asn 571B	2.47				
Leu 647B	3.71	Asn 631B	2.42				
Arg 664A	3.61	Asp 644B	1.94				
Arg 664B	3.40	Arg 664A	2.97				
Arg 664B	3.58						

Table S2. Binding interactions of LPHVLTPEAGAT-$(DOX)_2$ [(L-pep)-DOX_2] with FGFR2 Kinase domain (A and B are indicative of the A and B chains involved).

Hydrophobic Interactions		H-Bond Interactions		Salt Bridges		π-stacking	
Residue	Distance (Å)	Residue	Distance (Å)	Residue	Distance (Å)	Residue	Distance (Å)
Leu 487A	3.92	Glu 489B	3.29				
Leu 487A	3.41	Glu 489B	3.07				
Cys 491B	3.35	Cys 491B	3.13				
Cys 491B	3.77	Phe 492B	1.96				
Phe 492B	3.89	Gly 493A	2.27				
Phe 492B	3.72	Lys 517B	2.95				
Val 495B	3.67	Lys 520A	2.60				
Thr 524A	3.57	Asp 521A	2.33				
Leu 633A	3.73	Asp 522A	2.44				
		Asp 522A	2.24				
		Thr 524A	2.70				
		Asp 527A	1.71				
		Glu 534B	3.23				
		Ala 567A	1.98				
		Ala 567A	2.06				
		Asn 571A	1.94				

		Asn 571A	3.39				
		Arg 630A	1.87				
		Asp 644B	2.87				

Table S3. Binding interactions of ACSAG (A-pep) with FGFR2 Kinase domain (A and B are indicative of the A and B chains involved).

Hydrophobic Interactions		H-Bond Interactions		Salt Bridges		π-stacking	
Residue	Distance (Å)	Residue	Distance (Å)	Residue	Distance (Å)	Residue	Distance (Å)
Leu 487B	3.83	Phe 492B	2.81				
Cys 491B	3.69	Lys 517B	2.14				
Phe 492B	3.63	Asn 571B	2.24				
Val 495B	3.88	Arg 630B	2.27				
		Asp 644B	2.32				

Table S4. Binding interactions of ACSAG-DOX [A-pep-DOX] with FGFR2 Kinase domain (A and B are indicative of the A and B chains involved).

Hydrophobic Bonding		H-Bond Interactions		Salt Bridges		π-stacking	
Residue	Distance (Å)	Residue	Distance (Å)	Residue	Distance (Å)	Residue	Distance (Å)
Cys 491B	3.87	Cys 491B	2.32			Phe 492B	4.49
Phe 492B	3.86	Lys 517A	2.33				
Arg 664B	3.93	Asp 527A	1.73				
		Asp 626A	3.43				
		Arg 630A	2.32				
		Arg 630A	2.25				
		Arg 630B	3.19				
		Asn 631A	2.32				
		Asn 631A	2.30				
		Asp 644A	2.24				
		Asp 644B	2.39				
		Arg 664A	2.58				
		Arg 664A	2.30				

Table S5. Binding interactions of ACSAG-LPHVLTPEAGAT-GASCA [Trimer-Pep] with FGFR2 Kinase domain (A and B are indicative of the A and B chains involved).

Hydrophobic Interactions		H-Bond Interactions		Salt Bridges		π-stacking	
Residue	Distance (Å)	Residue	Distance (Å)	Residue	Distance (Å)	Residue	Distance (Å)
Pro 486A	3.84	Leu 487A	2.38	Lys 517A	4.74		
Leu 487A	3.50	Gly 490B	3.19				
Glu 489B	3.62	Val 495A	3.05				
Val 495A	3.36	Lys 517A	2.22				
Val 495A	3.69	Lys 520A	2.63				
Val 495A	3.56	Lys 520B	3.19				
Lys 520A	3.76	Asp 521B	3.12				
Asp 521B	3.50	Asp 522B	2.59				
Arg 630A	3.64	Asp 522B	2.45				
Leu 633A	3.63	Asp 527A	3.24				
Ala 643A	3.41	Asn 571A	2.91				
Leu 647A	3.68	Arg 630A	2.33				
Leu 647A	3.99	Arg 630A	2.35				
		Arg 630A	3.20				
		Asp 644A	2.31				
		Asp 644A	2.74				
		Asp 644A	2.82				
		Arg 664A	2.30				

Table S6. Binding interactions of (ACSAG-LPHVLTPEAGAT-GASCA)-$(DOX)_2$ [Trimer-Pep-$(DOX)_2$] with FGFR2 Kinase domain (A and B are indicative of the A and B chains involved).

Hydrophobic Interactions		H-Bond Interactions		Salt Bridges		π-stacking	
Residue	Distance (Å)	Residue	Distance (Å)	Residue	Distance (Å)	Residue	Distance (Å)
Pro 486A	3.73	Leu 487A	3.60	Lys 520B	3.50		
Leu 487A	3.73	Glu 489B	3.22				
Glu 489B	3.43	Glu 489B	3.08				
Glu 489B	3.94	Gly 490B	2.03				
Lys 517A	3.63	Gly 493A	2.55				
Arg 630A	3.65	Gly 493A	2.86				
Asp 644A	3.84	Gln 494B	2.02				
		Val 495B	2.69				
		Val 495B	2.60				
		Lys 520A	2.30				
		Lys 520B	3.41				
		Asp 522A	1.87				
		Asp 522A	2.77				
		Asp 522B	3.64				
		Asp 522B	3.20				
		Thr 524A	3.35				
		Asp 527A	2.29				
		Asp 527A	3.51				
		Asn 571A	3.27				
		Asn 571A	2.74				
		Asn 571B	2.67				
		Glu 574B	2.78				
		Glu 574B	2.93				
		Asp 626B	3.01				
		Arg 630A	3.29				
		Arg 630B	3.10				
		Arg 630B	3.08				
		Arg 664A	2.76				
		Arg 664A	3.60				
		Arg 664B	3.06				

Table S7. Binding interactions of Doxorubicin $(DOX)_2$ with FGFR2 Kinase domain (A and B are indicative of the A and B chains involved).

Hydrophobic Interactions		H-Bond Interactions		Salt Bridges		π-stacking	
Residue	Distance (Å)	Residue	Distance (Å)	Residue	Distance (Å)	Residue	Distance (Å)
Cys 491B	3.29	Cys 491B	2.26				
Phe 492B	3.84	Phe 492B	2.57				
Lys 520A	3.92	Gly 493B	2.36				
		Gly 493B	2.75				
		Lys 517B	2.79				
		Asp 527A	2.36				
		Asp 644B	2.97				

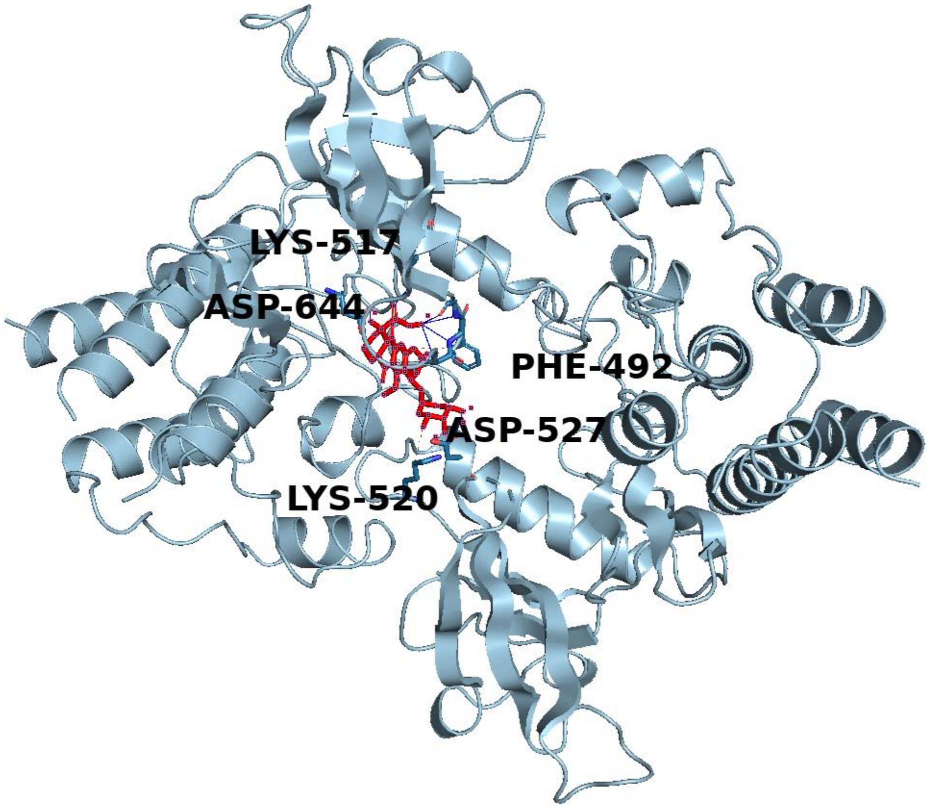

Figure S1. Molecular docking results showing binding interactions of DOX (doxorubicin) with FGFR2 kinase domain. DOX is represented in red.
